# Evaluation of SNP Genotyping in Alpacas Using the Bovine HD Genotyping Beadchip

**DOI:** 10.3389/fgene.2019.00361

**Published:** 2019-04-24

**Authors:** Manuel More, Gustavo Gutiérrez, Max Rothschild, Francesca Bertolini, F. Abel Ponce de León

**Affiliations:** ^1^Facultad de Zootecnia, Universidad Nacional Agraria La Molina, Lima, Peru; ^2^Department of Animal Science, Iowa State University, Ames, IA, United States; ^3^National Institute of Aquatic Resources, DTU-Aqua, Technical University of Denmark, Lyngby, Denmark; ^4^Department of Animal Science, University of Minnesota, Minneapolis, MN, United States

**Keywords:** alpaca, bovine, SNP, genotyping, polymorphic

## Abstract

Alpacas are one of four South American Camelid species living in the highlands of the Andes. Production of alpaca fiber contributes to the economy of the region and the livelihood of many rural families. Fiber quantity and quality are important and in need of a modern breeding program based on genomic selection to accelerate genetic gain. To achieve this is necessary to discover enough molecular markers, single nucleotide polymorphisms (SNPs) in particular, to provide genome coverage and facilitate genome wide association studies to fiber production characteristics. The aim of this study was to discover alpaca SNPs by genotyping forty alpaca DNA samples using the BovineHD Genotyping Beadchip. Data analysis was performed with GenomeStudio (Illumina) software. Because different filters and thresholds are reported in the literature we investigated the effects of no-call threshold (≥0.05, ≥0.15, and ≥0.25) and call frequency (≥0.9 and =1.0) in identifying positive SNPs. Average GC Scores, calculated as the average of the 10% and 50% GenCall scores for each SNP (≥0.70) and the GenTrain score ≥ 0.25 parameters were applied to all comparisons. SNPs with minor allele frequency (MAF) ≥ 0.05 or ≥ 0.01 were retained. Since detection of SNPs is based on the stable binding of oligonucleotide probes to the target DNA immediately adjacent to the variant nucleotide, all positive SNP flanking sequences showing perfect alignments between the bovine and alpaca genomes for the first 21 or 26 nucleotides flanking the variant nucleotide at either side were selected. Only SNPs localized in one scaffold were assumed unique. Unique SNPs identified in both reference genomes were kept and mapped on the Vicugna_pacos 2.0.2 genome. The effects of the no-call threshold ≥ 0.25, call frequency = 1 and average GC ≥ 0.7 were meaningful and identified 6756 SNPs of which 400 were unique and polymorphic (MAF ≥ 0.01). Assignment to alpaca chromosomes was possible for 292 SNPs. Likewise, 209 SNPs were localized in 202 alpaca gene loci and 29 of these share the same loci with the dromedary. Interestingly, 69 of 400 alpaca SNPs have 100% similarity with dromedary.

## Introduction

Alpacas are an important animal resource living in the highland areas of the Andes. They provide fiber, skins, meat and manure for agricultural production and, along with llamas, are a cornerstone of cultural heritage. Peru hosts about 85% of the worldwide alpaca population of which 80% belong to the Huacaya type, 12% to the Suri type and 8% are intermediate Ministerio de Agricultura y Riego [MINAGRI] (2017). Alpacas are kept mainly for fiber production and meat is a secondary product. Production of alpaca fiber contributes to the regional economy and is in high demand by the textile industry. In 2015 fiber production reached 4,478t at national level, of which 90% was for export market and 10% for the Peruvian market. Individual alpaca breeding program initiatives by private companies, NGOs and farmer cooperatives aimed to improve fiber quality by reducing fiber diameter. Much could be gained with the application of genomic selection to accelerate genetic gain. However, there is still limited information about the alpaca genome organization and a paucity in developing molecular markers necessary for the application of modern animal selection programs.

Several advances in the understanding of the organization of the alpaca genome have occurred in the last decade. The alpaca genome has been sequenced by two separate research groups at a depth of ∼22X (Warren et al., 2013) and 72.5X (Wu et al., 2014). Their corresponding genome assemblies are publicly available. Similarly, chromosomal identification of syntenic regions between human, bovine and camelid by Zoo-FISH have allowed the preliminary assignment of alpaca genome scaffolds to specific alpaca chromosomes (Balmus et al., 2007). Avila et al. (2014) extended the latter, by developing the first cytogenetic map containing 230 chromosomally localized molecular markers and genes. However, there is still a limited number of available molecular markers (Pérez-Cabal et al., 2010; Paredes et al., 2014) and subsequently a very limited number of association studies of genetic markers to production traits in alpacas have been performed (Guridi et al., 2011; Paredes et al., 2014; Chandramohan et al., 2015). Therefore the identification of additional single nucleotide polymorphisms (SNPs) is necessary to improve the SNP coverage across the genome (Munyard et al., 2009), to increase the possibility of identifying linkage disequilibrium between markers and therefore to perform genome-wide association analyses with production traits (Hayes and Goddard, 2010; Dekkers, 2012).

The lack of SNP microarrays for non-model organisms has led to test commercially available SNP microarrays of closely related species to discover common SNPs. Slate et al. (2009) have reviewed alternatives to cross-species application of commercial SNP chips for SNP discovery. Most are labor intensive, high cost, and yield low numbers of SNP in comparison to genotype-by-sequencing (GBS) methods that yield abundant species-specific SNPs at low cost (Miller et al., 2012). However, GBS is prone to higher calling rate errors than genotyping with SNP chips because it relies on pooling random sequence information from several individuals and loci increasing the probability of low coverage for some individual/locus combinations. SNP chips, on the other hand, have the advantage that each locus is present multiple times in the chip and genotypes are called by averaging over all of the individual calls per SNP, resulting in accurate genotype calls (Oliphant et al., 2002). Another advantage of SNP chips is the evaluation of the same loci across all individuals per experiment, which is possibly more difficult to achieve with GBS within experiment and across experiments. The latter is because GBS methods are based on generating sequencing libraries with restriction enzyme digested DNA that leads to variance representation of loci among individuals. Some of these limitations could be overcome by genotype imputation (Li et al., 2009) if a reference panel of genotypes is available. The latter is mostly lacking for non-model organisms.

The main purpose in using commercially available SNP chips is the identification of conserved cross species SNPs, reported in the literature as cross-species amplification, cross-amplification or cross-species genome-wide arrays. For example, Malhi et al. (2011) genotyped seven old world monkey species using an Illumina Golden Gate Array of *Macaca mulatta*, a closely related species, reporting 173 polymorphic SNPs. Likewise, Miller et al. (2012) studied the relationship between the successful applicability of cross-species SNP microarrays and evolutionary time using OvineSNP50, BovineSNP50 and EquineSNP50 BeadChips to identify SNPs in target wild species. They reported that the call rate decreased ∼1.5% per each million years of divergence time between species and the polymorphism retention of SNPs declined exponentially leveling off after about 5 Myr of divergence. Moreover, SNP genotyping in wood bison, plains bison and European bison (Pertoldi et al., 2010), scimitar-horned and Arabian oryx (Ogden et al., 2012) were performed using the Illumina BovineSNP50 BeadChip, reporting 1524, 1403, 929, 148, and 149 polymorphic SNPs, respectively. SNP genotyping in dromedary was performed using the Illumina Bovine 777K SNP BeadChip and the Illumina Ovine 600K SNP BeadChip microarrays (Bertolini et al., 2017), reporting 29900 bovine and 14179 ovine SNPs successfully genotyped.

Kharzinova et al. (2015) also reported that 43.0 and 47.0% of all SNPs in the Illumina BovineSNP50 BeadChip and the Illumina OvineSNP50 BeadChip, respectively, could be genotyped in reindeer. In addition, Haynes and Latch (2012) and Moravèíková et al. (2015) reported that 38.7 and 53.89% of the SNPs in the Illumina Bovine SNP50 BeadChip, respectively, were identified in cervids, in at least 90% of individuals, despite 25.1–30.1 million years divergence between Bovidae and Cervidae (Hassanin and Douzery, 2003). Furthermore, Hoffman et al. (2013) reported that 19.2% of all SNPs of the Illumina CanineHD BeadChip could be genotyped in seals, and reported 173 polymorphic SNPs despite a phylogenetical divergence time of around 44 million years. Therefore, the use of SNP microarrays of species with well-studied genomes have the potential to identify SNPs in related and widely diverged species.

**FIGURE 1:**
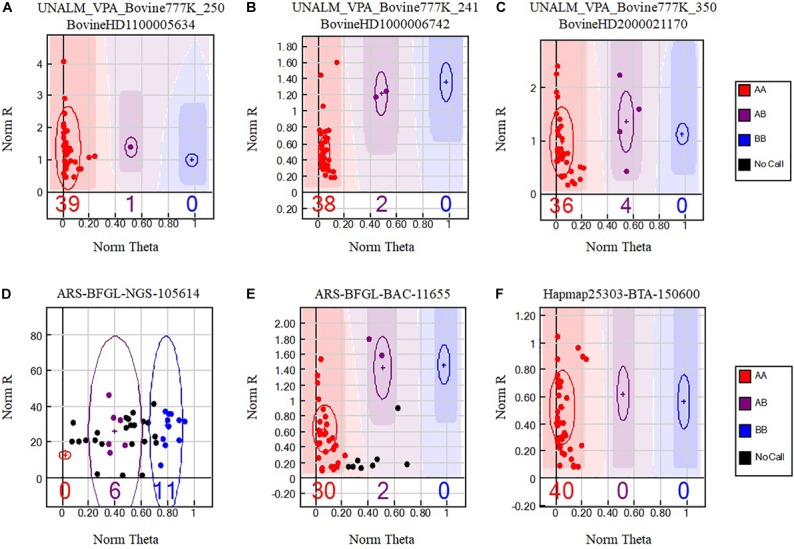
Single nucleotide polymorphisms (SNPs) from the BovineHD Genotyping Beadchip that cross-amplify in alpacas. Genotypes are called for each sample (dot) by their signal intensity (Norm R, Y-axis) and Allele Frequency (Norm Theta, X-axis) relative to canonical cluster positions (dark shading) for a given SNP marker (red = AA, purple = AB, blue = BB). Black points represent no call samples. Polymorphic SNP genotype cluster patterns **(A–C)** selected with Method VI are presented. Difficult to interpret genotype cluster patterns **(D,E)** that were not retained are also shown and a monomorphic genotype cluster pattern **(F)** present among the 6756 positive SNPs.

Interestingly, all of the reported cross species analysis used different versions of GenomeStudio (Illumina, United States) and were not comparable as each research group gave different weights to parameters used to generate their genotyping results. Haynes and Latch (2012) and Moravèíková et al. (2015) used a Call Frequency (Call Freq) ≥ 0.9 while Pertoldi et al. (2010) and Kharzinova et al. (2015) used a Call Freq = 1. Call Frequency was calculated as the number of genotype calls divided by the sum of no-calls and calls for each SNP. Lower Call-Frequency increases accuracy (Oliphant et al., 2002). Aiming at increasing the stringency of the analysis other research groups considered GenTrain score ≥ 0.25 (Hoffman et al., 2013) or the average GC score (average GC) ≥ 0.7 (Bertolini et al., 2017). The GenTrain score takes into account the quality and shape of the genotype clusters (Figure 1) and their relative distances from one another for each SNP while the average GC is calculated for each SNP as the average of the 10^th^ percentile and 50^th^ percentile of the distribution of GenCall scores.

Given the above experiences, the aim of this study was to evaluate SNP genotyping in alpacas using the BovineHD Genotyping Beadchip (Illumina, United States), in spite of 42.7 million years of evolutionary divergence between these two species (Wu et al., 2014) and to evaluate the different analysis methods reported in the literature.

## Materials and Methods

### DNA Samples and Genotyping

Blood samples from 40 Huacaya type alpacas (4 females and 36 males) were collected by venipuncture and transferred to FTA cards. Organic DNA extraction and genotyping was done at Neogen-Geneseek laboratories (United States). Samples were genotyped using the BovineHD Genotyping Beadchip (777962 SNPs, Illumina). The sample set of unrelated animals originated from two geographical distinct Andean regions and from two separate alpaca farms within region, Chagas Chico and San Pedro de Racco in the central Andes and INCA TOPS S.A. and MICHELL & CIA S.A in the most southern Andes. The number of animals used for this study was determine to be the minimum necessary to identify SNPs with minor allele frequency (MAFs) = 0.0125 that will allow to observe at least one heterozygous genotype per sample and per SNP.

### Data Analysis

Bioinformatics analysis was performed at the Universidad Nacional Agraria La Molina, Lima, Peru. The software GenomeStudio 2011.1 (Genotyping module version 1.9.4, Illumina, United States) was used to analyze the genotyping reports. GenomeStudio normalizes the intensities of signals for each locus and assigns a cluster position for each sample. Three parameters, no-call threshold, call frequency, and average GC were evaluated. No-call threshold or GenCall score cutoff is a quality metric calculated for each genotype (data point) and ranges from zero to one. GenCall scores decrease in value the further they are from the center of the cluster to which they are associated (Figure 1A–C). A no-call threshold of 0.15 is normally used for analysis of Infinium data when genotyping the same species. Hence, genotypes with GenCall scores less than 0.15 are not assigned genotypes because of being far away from the center of a cluster and therefore are categorized as a no call for the locus (Figure 1D,E; black dots). Call Frequency is calculated as the number of genotype calls divided by the sum of no-calls and calls for each SNP. The average GC is the simple average of the 50%GC and the 10%GC scores calculated for each SNP, where the 50%GC score represent the 50^th^ percentile of GenCall scores across all called genotypes and the 10%GC score represents the 10^th^ percentile. The parameters of call frequency, 50%GC and 10%GC evaluate the quality and performance of DNA samples within an experiment. Our analysis was performed using seven combinations of values for the latter three parameters. These seven combinations are labeled as Methods and are presented in Table 1. These methods aimed at comparing the effect of call frequency 0.9 and 1 under different no-call threshold values of ≥0.05 (Method I and Method II), ≥0.15 (Method III and Method IV) and a more stringent no-call threshold ≥0.25 (Method V, Method VI. and Method VII; Hoffman et al., 2013) in selecting SNPs. The average GC score calculated for each SNP ranks the genotype call signal from 0 (bad) to 1 (good) (Bertolini et al., 2017). We have used an average GC score value of ≥0.7 for all methods except Method VII. Similarly, a GenTrain score ≥ 0.25 (Hoffman et al., 2013) was used for all methods evaluated. The GenTrain score, calculated for each SNP by GenomeStudio, takes into account the shape of the genotype cluster and their relative distance from one another within a cluster. For all methods, positive SNPs with MAF ≥ 0.01 were retained as polymorphic SNPs.

**Table 1:** Parameter values used for each method of analysis.

Parameter	Method I	Method II	Method III	Method IV	Method V	Method VI	Method VII
No-call threshold	≥0.05	≥0.05	≥0.15	≥0.15	≥0.25	≥0.25	≥0.25
Call frequency	≥0.9	1	≥0.9	1	≥0.9	1	1
Average GC	≥0.7	≥0.7	≥0.7	≥0.7	≥0.7	≥0.7	*
GenTrain score	≥0.25	≥0.25	≥0.25	≥0.25	≥0.25	≥0.25	≥0.25

*^∗^In Method VII, average GC ≥ 0.7 parameter was not applied.*

### Alignment of Flanking Sequence of Alpaca Positive Bovine SNPs With Reference Alpaca Genomes

To confirm that discovered alpaca SNPs were indeed polymorphic, two alpaca genome assemblies [Vicugna_pacos-2.0.2, GCA_000164845.3, with 22X coverage and assembled into 3374 scaffolds (KB632434-KB635807); and Vi_pacos_V1.0, GCA_000767525.1, with 72.5X coverage and assembled into 4322 scaffolds (KN266727–KN271048)] were used to align flanking sequences of alpaca positive polymorphic bovine SNPs for each method under comparison.

Microarray genotyping of SNPs result from hybridizing denatured fragments of the DNA being genotyped (target DNA) to 50 bp long SNP probes anchored on beads within a microarray chip. We hypothesize that for the identification of positive SNPs at least the first 21 to 26 nucleotides flanking the polymorphic nucleotide of the probe would need to be 100% similar to the target DNA, allowing for the rest of the probe and target sequences less than perfect similarity while permitting the priming extension of the probe fragment by the polymerase. This latter hypothesis is supported in part by Sechi et al. (2009) who reported that increased sequence divergence (mismatches) toward the 3′ end of the probe immediately flanking the variant nucleotide would have the greatest destabilizing hybridization effect resulting in no calls. Therefore, the 5′ end sequences used for BLAST analysis started at the 20^th^ or 25^th^ nucleotide 5′ to the polymorphic nucleotide and ended with allele A or allele B of the polymorphic nucleotide at the 3′ end. Conversely, the 3′ end flanking sequences were read on the negative DNA strand, started at the allele A or allele B of the polymorphic nucleotide, and ended at the 20^th^ and 25^th^ nucleotide at its 5′ end. These alignments were performed using the BLAST (blastn-short task) software of the Galaxy Platform hosted at the Minnesota Supercomputing Institute (University of Minnesota). SNPs flanking sequences that showed perfect alignments were selected, and a list with these SNPs was generated for each alpaca reference genome. Only SNPs that were unique and detected in both reference genomes were retained. Since only 100% sequence similarity between a positive bovine SNP and the alpaca genome was observed for the first 20 or 25 nucleotides flanking the variant nucleotide, the rest of the sequence to generate the 101 nucleotide sequence of alpaca SNPs was retrieve from the Vicugna_pacos 2.0.2. Hardy–Weinberg equilibrium, based on genotype distributions for each SNP, was evaluated with Genpop (Rousset, 2017) and ChiTest_p100 (Illumina Proprietary, 2008). Finally, these SNPs were assigned to alpaca chromosomes based on chromosome syntenies between cattle and camelid as described by Balmus et al. (2007) and scaffold assignments to chromosomes as described by Avila et al. (2014). Since, the phylogenetic analysis done by Kadwell et al. (2001) suggested a Latin name change for alpacas to *Vicugna pacos*; we have adopted the acronym VPA for alpaca chromosomal naming in this manuscript.

### Identification of Nearest Genes to Alpaca Polymorphic SNPs and Alpaca/Dromedary SNPs

The Vicugna_pacos 2.0.2 reference genome was used to identify the most proximal gene to each polymorphic SNPs. A list of these genes was develop and used for gene ontology (GO) analysis^1^ for biological process GO terms. Similarly, we aligned alpaca polymorphic SNP sequences to the dromedary reference genome (PRJNA234474_Ca_dromedarius_V1.0, GCF_000767585.1) to assess SNP sequence conservation between alpaca and dromedary.

## Results

The number of bovine SNPs yielding positives signals are reported in Table 2 for each of the analysis methods as described in Table 1. As expected, the parameters call frequency and no-call threshold had an inverse effect on the total number of positive SNPs, decreasing in number as no-call threshold and call frequency increased. Out of the 777962 SNPs analyzed 68.1, 47.3, and 33.7% were detected with a call frequency of 0.9 (Methods I, III, and V), while 14.3, 5.1, and 3.0% were detected with a call frequency of 1 (Methods II, IV, and VI, respectively). However, when average GC ≥ 0.7 was applied, a further reduction of positive SNPs was observed with 2.9, 1.5, 3.2, 1.1, 3.3, and 0.9% for Methods I, II, III, IV, V, and VI, respectively.

**Table 2:** Number of positive SNPs by method.

Parameter of analysis	Method I	Method II	Method III	Method IV	Method V	Method VI	Method VII
	No-call threshold ≥0.05		No-call threshold ≥0.15		No-call threshold ≥0.25		
	Call freq ≥ 0.9	Call freq = 1	Call freq ≥ 0.9	Call freq = 1	Call freq ≥ 0.9	Call freq = 1	Call freq = 1
Call frequency	530106	111471	368001	39279	262506	23429	23429
Average GC (≥0.7)	22437	11364	24979	8232	25609	6756	∗
GenTrainScore (≥0.25)	22437	11364	24979	8232	25609	6756	23429
MAF (≥0.01)	22435	11364	24962	8232	25563	6756	23427
MAF (≥0.05)	1970	898	1724	430	1467	274	2044

*^∗^In Method VII, average GC ≥ 0.7 parameter was not applied.*

The percentage decrease in positive SNPs observed between Methods I and II is 21.0%, Methods III and IV is 10.7%, and Methods V and VI is 8.9%. Hence, the percentage difference of positive SNPs within a no-call threshold value decreases as the call frequency increases. However, this decrease is less pronounced as the no-call threshold increased. The differences of detected SNPs between Method I and Method II (53.8%), Method III and Method IV (42.3%) and, Method V and Method VI (30.7%), suggested that the effect of call frequency decreases when the no-call threshold increases.

The comparison of results between Methods VI and VII illustrate the effect of the average GC parameter. The number of retained SNPs in Method VI is 6756 representing a reduction of 71.2% when compared to Method VII. Hence, the effect of the average GC parameter was important in reducing the number of false positive SNPs. The GenTrain score ≥ 0.25 did not show any effect on the number of retained SNPs when the average GC ≥ 0.7 was applied. In Supplementary Table S1 we present the minimum, maximum, mean, and standard deviation scores of average GC and GenTrain score observed for each method. However, we did not test if these latter two parameters are interchangeable.

Significant reduction in the number of SNPs retained was observed when SNPs with MAF ≥ 0.05 are selected going from 91% reduction for Method I to 96% for Method VI. Under the conditions of our analysis, Method VI showed the highest stringency and identified 6756 SNPs with MAF ≥ 0.01.

In Table 3 we present results obtained from the alignment of all retained SNPs, with MAF ≥ 0.05, to both alpaca reference genomes. Likewise, similar analysis is presented for Method VI for SNPs with MAF ≥ 0.01.

**Table 3:** Number of positive bovine SNPs aligned to the alpaca reference genomes.

Reference genome	Method I	Method II	Method III	Method IV	Method V	Method VI	Method VII	Method VI
				MAF ≥ 0.05				MAF ≥ 0.01
**Vicugna_pacos-2.0.2**								
Aligned to more than one scaffold	10	5	7	3	7	1	9	33
Unique SNPs	94	45	72	23	67	18	154	467
**Vi_pacos_V1.0**								
Aligned to more than one scaffold	10	6	6	3	6	1	11	30
Unique SNPs	95	41	72	21	68	17	146	466
**SNPs common to both reference genomes**	79	36	62	18	59	16	129	400
**SNPs with predicted chromosomal localization**	57	29	49	15	45	13	98	292

Out of all the polymorphic SNPs with MAF ≥ 0.05 presented in Table 2, 5.3, 5.6, 4.6, 6.1, 5.0, 6.9 and 8.0%, were aligned to the Vicugna_pacos-2.0.2 genome assembly for Methods I, II, III, IV, V, VI, and VII, respectively. Moreover, 5.3, 5.2, 4.5, 5.6, 5.0, 6.6, and 7.7% were aligned to the Vi_pacos_V1.0 genome assembly for Methods I, II, III, IV, V, VI, and VII, respectively. Some of the SNPs with MAF ≥ 0.05 presented in Table 2 were identified in more than one scaffold and a few were repeated within a single scaffold. Therefore, only 4.0, 4.0, 3.6, 4.2, 4.0, 5.8, and 6.3% were unique and were common to both genomes, for Methods I, II, III, IV, V, VI, and VII, respectively.

From the unique SNPs identified for each method we could only assigned 57, 29, 49, 15, 45, 13, and 98 SNPs to alpaca chromosomes for Methods I, II, III, IV, V, VI, and VII, respectively. These assignments are based on chromosome homology between cattle and camelid described by Balmus et al. (2007) or based on the cytogenetic map information developed by Avila et al. (2014).

**FIGURE 2:**
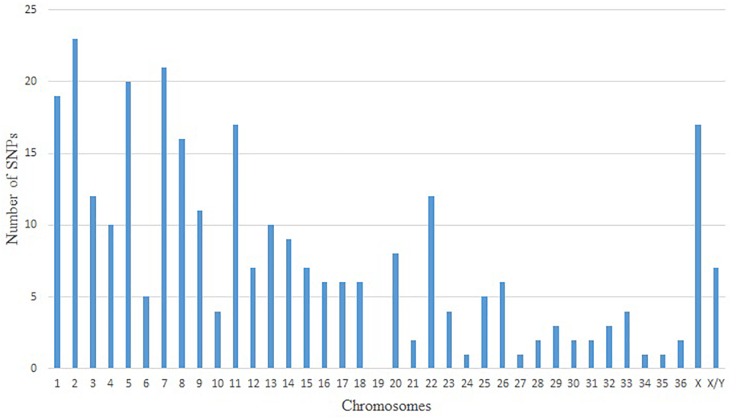
Distribution of positive and unique SNPs in alpaca chromosomes by predicted localization.

Since the no-call threshold, call frequency, and average GC parameters were more stringent for Method VI, we selected the 400 unique SNPs with MAF ≥ 0.01 common to both reference genomes as a new set of alpaca SNPs identified in this study. The MAFs of these SNPs ranged from 0.0125 to 0.075 of which 342 SNPs had a MAF = 0.0375 (Supplementary Table S2) and only seven SNPs were not in Hardy–Weinberg equilibrium.In Figure 1 we present three examples of selected unique and three unselected SNPs obtained with Method VI. All 400 SNPs showed the classical genotype cluster pattern expected from polymorphic SNPs (Figure 1A–C) while the unselected showed difficult to interpret genotype cluster patterns (Figure 1D,E) with the exception of monomorphic SNPs (Figure 1F). Of the 400 unique SNPs, 292 SNPs were assigned to alpaca specific chromosomes (Figure 2 and Supplementary Table S2). Interestingly, no SNP was assigned to VPA19.

Of the 400 polymorphic 209 were localized within 202 annotated alpaca genes (Vicugna_Pacos-2.0.2) and 69 of 400 SNPs showed perfect flanking alignment of 101 nucleotides between alpaca and dromedary. Moreover, 29 SNPs of the 69 SNPs were localized in similarly annotated dromedary and alpaca genes (Supplementary Table S3). The ontology analysis of the 202 annotated genes displays five GO terms that were enriched for genes at the polymorphic SNPs. The five GO terms identified were, (1) positive regulation of synaptic transmission (10 genes), (2) cell morphogenesis (20 genes), (3) cell adhesion (24 genes), (4) generation of neurons (35 genes), and (5) regulation of multicellular processes (52 genes). The majority of these genes are involved in biological developmental processes.

## Discussion

The application of genome wide association studies (GWASs) studies to alpacas will only be possible when enough SNPs are identified to provide a reasonable coverage of their genome. This study tested a cross hybridization approach for the identification of conserved polymorphic cattle/alpaca SNPs using the available BovineHD Genotyping Beadchip. The assessment of combination of scores for no-call threshold, call frequency and average GC yielded an optimum method that identified 400 conserved polymorphic SNPs. However, these latter SNPs are affected by ascertainment bias because of our small sample population and lack of information as to whether the SNPs originate from coding or non-coding regions that influence their minor allele frequencies. This small sample population will allow to detect SNPs with MAF ≥ 0.0125 therefore rare SNPs will not be represented. It has been suggested by Hoffman et al. (2013) that SNPs cross-amplified from high-density arrays might be enriched for conserved genomic regions retaining ancestral polymorphisms. However, the commercially available Bovine HD SNP chip we used in this study was designed to provide uniform genome coverage with evenly spaced SNPs and therefore it can be inferred that our discovered SNPs are selectively neutral. Nielsen (2004) provides a thorough review on ascertainment bias for SNP data.

One measure of genotyping success is the SNP conversion rate defined as the proportion of all genotyped SNPs showing clear genotyping clusters (Figure 1A–C). Our conversion rate was very low (0.008%) and is in line with observed conversion rates for cross hybridization genotyping experiments (Hoffman et al., 2013). The 400 polymorphic SNPs remain to be validated by genotyping a different and larger alpaca population sample.

### Data Analysis

The percentage of SNPs identified in at least 90% of samples by Method I and Method III was higher, 68.1 and 47.3%, respectively, than those SNPs found in the genotyping of deers (38.7%, Haynes and Latch, 2012). Moreover, the percentage of SNPs in Method I was also higher than those SNPs reported in cervids (53.9%, Moravèíková et al., 2015) using the Illumina BovineSNP50 Bead Chip. However, the percentage of SNPs observed using a more stringent no-call threshold (Method III) was less than these reports.

The percentages of SNPs identified with call frequency = 1 in Methods II (14.3%), IV (5.1%), and VI (3.0%) were less than those found in the genotyping of bisons (97.0%, Pertoldi et al., 2010) using the Illumina BovineSNP50 Bead Chip, and reindeers (43.0%, Kharzinova et al., 2015) using the Illumina BovineSNP50 v2. Bead Chip.

The percentages of SNPs identified with call frequency = 1 and selected based on their average GC ≥ 0.7 in Methods II (1.5%), IV (1.0%), and VI (0.9%) are less than those found in the genotyping of camels (3.8%, Bertolini et al., 2017) using the Illumina Bovine 777K SNP BeadChip. This could be due to higher heterogeneity of the dromedary sample in comparison to our alpaca sample set and/or in part determined by higher false positives identified in the dromedary-bovine cross hybridization experiments as stated by Bertolini et al. (2017).

The effects of the no-call threshold ≥ 0.25, call frequency = 1 and average GC ≥ 0.7 were significant in reducing the number of positive SNPs. However, under the conditions imposed by our analysis the use of GenTrain score threshold ≥ 0.25 (Hoffman et al., 2013) did not have any effect on the identification of positive SNPs in all methods at an average GC ≥ 0.7. However, it cannot be discarded that the GenTrain score threshold ≥ 0.25 might have a similar effect if it is used in substitution of the average GC ≥ 0.7 parameter.

The percentage of polymorphic SNPs in Methods II (1.5%), IV (1.1%), and VI (0.9%) is less than those found in the genotyping of deers (2%, Haynes and Latch, 2012), bisons (4.1%, Pertoldi et al., 2010), cervids (2.8%, Moravèíková et al., 2015), reindeers (2.3%, Kharzinova et al., 2015), and camels (3.6%, Bertolini et al., 2017). When a call frequency of 0.9 was used [Methods I (2.3%), III (3.2%), and V (3.3%)], the percentage of retained SNPs was higher in comparison to those reported by Haynes and Latch (2012); Kharzinova et al. (2015), and Moravèíková et al. (2015). In addition, the number of SNPs with MAF ≥ 0.05 were rare among the 40 samples analyzed.

Method VI identified 6756 SNPs with MAF ≥ 0.01 of which 400 showed perfect flanking alignment of 20 or 25 nucleotides adjacent to the polymorphic nucleotide and were further analyzed by manually observing their genotype cluster distributions where at least one sample was identified as heterozygous for each SNP. When applying the exponential polymorphic decay function developed by Miller et al. (2012) to our findings, the expected percentage of polymorphic SNPs is 0.000515% and our observed 6756 SNPs with MAF ≥ 0.01 identified with Method VI represent 0.008684%, which is 16.5 times higher than expected. However, this observed number of SNPs could represent an overestimate since we have not ascertained the polymorphic status of each of these putative SNPs. However, the 400 polymorphic SNPs reported in this study represent 0.000514%, which is similar to the calculated expected percentage of polymorphic SNPs obtained with the exponential decay function formula developed by Miller et al. (2012). Examples of polymorphic SNPs discovered in this study are presented in Figure 1A–C, showing the genotype cluster distributions of positively identified SNPs. For illustration purposes, we also present cluster distributions of two SNPs that are difficult to interpret and were not retained (Figure 1D,E) with our analysis as well as a monomorphic SNP (Figure 1F). The so-called monomorphic SNPs, represent alpaca DNA fragments that have hybridized to specific probes in the SNP chip and are homozygous for the A or the B alleles in the sample population. These monomorphic SNPs could also be referred as false negatives. Monomorphic SNPs could very well be polymorphic SNPs if a larger sample set or a different sample set is used.

Only 292 out of the 400 polymorphic SNPs were mapped to alpaca chromosomes and 108 (27%) could not be assigned to chromosomes with available indirect methods (Balmus et al., 2007; Avila et al., 2014). The absence of SNPs assigned to VPA19 and the low number of SNPs (≥5) assigned to 14 other chromosomes is difficult to explained with our available data. In this study, all SNPs identified using Method VI were located across all bovine chromosomes (Supplementary Figure S1). Bertolini et al. (2017) also reported this latter distribution for dromedary SNPs. In this study, of SNPs identified by less stringency methods (Method I and Method III) localized one bovine SNP (BovineHD1300018765) on VPA19. Hence, we believe that the observed distribution of SNPs across chromosomes is due to the stringency applied in Method VI and our inability to chromosomally assigned 27% of the identified SNPs based on the level of resolution of the methods used, in this study, to infer alpaca chromosomal assignments.

A comparison of the 400 SNP sequences between alpaca and dromedary identified 209 of the 400 SNPs to be localized within 202 annotated alpaca genes (Vicugna_Pacos-2.0.2) and 69 SNPs showed perfect flanking alignment of 101 nucleotides between alpaca (Vicugna_Pacos-2.0.2) and dromedary (PRJNA234474_Ca_dromedarius_V1.0, GCF_000767585.1). Moreover, 29 SNPs out of the 69 SNPs were localized in similarly annotated dromedary and alpaca genes (Supplementary Table S3). An ontology analysis of the 202 annotated gene display five GO terms were identified as enriched for genes at the polymorphic SNPs that were Bonferroni corrected for *P* < 0.05. The five GO terms identified were positive regulation of synaptic transmission (10 genes), cell morphogenesis (20 genes), cell adhesion (24 genes), generation of neurons (35 genes), and regulation of multicellular processes (52 genes). The majority of these genes are involved in biological developmental processes. It is possible that for this latter reason they exhibit sequence conservation between alpaca and bovine that would explain the conserved retention of polymorphic SNPs at these loci. However, because of our small sample size and small number of genes associated to polymorphic SNPs, the latter analysis should be treated with caution.

## Conclusion

In spite of 42.7 million years of evolutionary divergence between cattle and alpacas (Wu et al., 2014), the application of the cross hybridization approach for the identification of polymorphic alpaca SNPs, based on the use of the BovineHD Genotyping Beadchip (Illumina), was successful. The comparison of different filtering methods indicated that no-call threshold, call frequency and average GC are important parameters to consider for the successful identification of polymorphic SNPs in cross hybridization experiments. Based on our results, the filters of no call threshold ≥ 0.25, call frequency = 1, average GC ≥ 0.7, and GenTrain score ≥ 0.25 are recommend for detection of SNPs in non-model species. The application of these filters allowed the identification of 6756 alpaca SNPs of which 400 are polymorphic and 292 SNPs were assigned to alpaca chromosomes. Further, 209 SNP were localized in 202 alpaca gene sequences and 29 of these were also located at similar gene loci in dromedary. Of the 400 alpaca SNPs, 69 shared 100% percent sequence similarity to dromedary. Our results represent a significant increase in polymorphic molecular markers for alpaca at this moment and indicates that investing in discovering SNPs by GBS or by sequencing reduced representation libraries of a larger number of samples would be necessary to generate an alpaca SNP chip for the successful application of GWAS to this species.

## Ethics Statement

The Universidad Nacional Agraria La Molina has recently established an Ethics Committee for Scientific Research by University Resolution No. 0345-2018-CU-UNALM of October 22, 2018 which has not initiated its operations as of yet. However, we have a letter signed by the Dean of the college of Animal Sciences corroborating that the protocol used for blood collection titled “Collection of Blood for FTA cards” is of conventional application and it follows the requirements of the National Act No. 30407 “Ley de Proteccion y Bienestar Animal” (Act for the Protection and Well-being of Animals).

## Author Contributions

FPL and MR conceived the study. MM, GG, and FPL participated in data analysis. MM and FPL co-wrote the manuscript. GG and FPL supervised the study. MR and FB reviewed and corrected the manuscript. All authors read and approved the manuscript.

## Conflict of Interest Statement

The authors declare that the research was conducted in the absence of any commercial or financial relationships that could be construed as a potential conflict of interest.

## Footnotes

1 geneontology.org
