# Supplementary figures and images for: Evaluation of SNP Genotyping in Alpacas Using the Bovine HD Genotyping Beadchip
Source: Front Genet. 2019 Apr 24;10:361. doi: 10.3389/fgene.2019.00361 (PMC6492526; doi:10.3389/fgene.2019.00361)

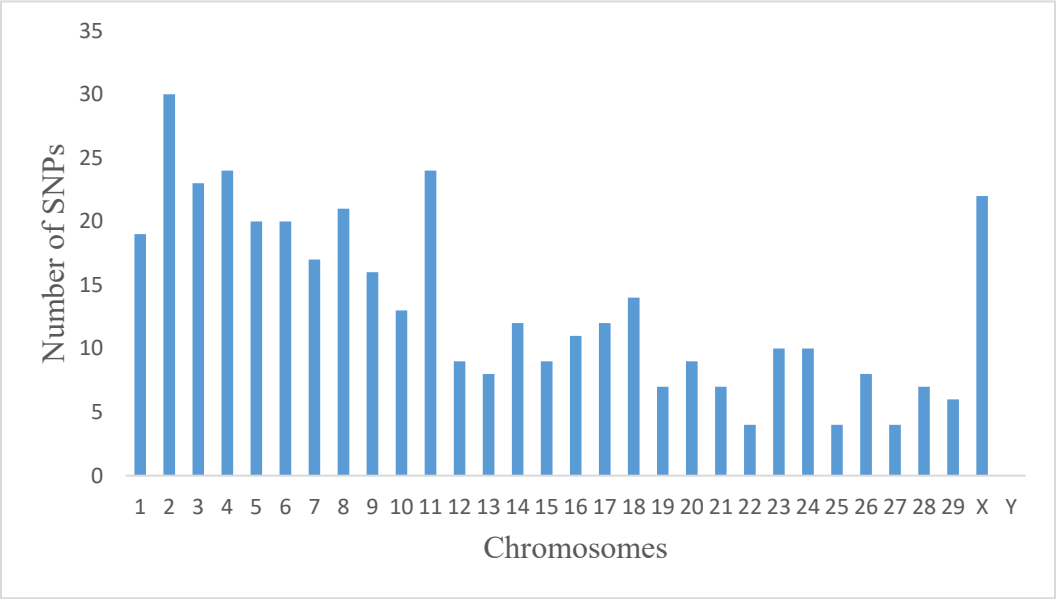

**Figure 1S. Distribution of positive and unique alpaca polymorphic SNPs on bovine chromosomes**

Supplement: Supplementary file 4 [file Image_1.pdf]
